# Response of Benthic Foraminifera to Cadmium Pollution Assessed via Morphological and Metabarcoding Analyses

**DOI:** 10.3390/microorganisms14051122

**Published:** 2026-05-15

**Authors:** Ling Qiao, Yuqi Wu, Jianping Zhao, Ye Chen, Jianglin Li, Qing Hao, Yuanming Guo, Tiejun Li

**Affiliations:** 1 Key Laboratory of Sustainable Utilization of Technology Research for Fishery Resource of Zhejiang Province, Zhejiang Marine Fisheries Research Institute, Zhoushan 316021, China; qiaoling1990123@126.com (L.Q.);; 2 Fishery College, Zhejiang Ocean University, Zhoushan 316000, China; 3 Pinghu City Marine (Island) Resources Service Center, Pinghu 314200, China

**Keywords:** benthic foraminiferal community, eRNA, eDNA, test formation, culture experiment

## Abstract

Benthic foraminifera are effective indicators of heavy metal contamination in marine ecosystems. Traditional methods for benthic foraminiferal identification and biodiversity assessment rely predominantly on stereomicroscopic analysis. However, this approach is time-consuming, labor-intensive, and cannot effectively identify small or morphologically similar species. In this study, we aimed to enhance the utility of benthic foraminifera as bioindicators. To this end, we investigated the responses of benthic foraminiferal communities to varying concentrations of Cd under controlled laboratory conditions using both morphological assessments and metabarcoding analyses. Cd exposure reduced the abundance of benthic foraminifera. High Cd concentrations led to Cd enrichment in foraminiferal tests and altered the contents of other elements. *Quinqueloculina*, *Ammonia*, and *Miliammina* exhibited tolerance to Cd, whereas *Parasorites* and *Ovammina* were more sensitive. This study provides an effective approach for evaluating the short-term effects of heavy metal pollution on benthic foraminiferal communities.

## 1. Introduction

Cd is a non-essential trace metal that poses serious ecological and human health risks owing to its persistence, high toxicity, and tendency to bioaccumulate [1,2]. Although natural background concentrations of Cd in seawater are typically low (0.001–0.1 μg/L), anthropogenic inputs from agriculture, industry, and coastal development have substantially increased Cd levels in many coastal areas [3,4]. Cd exerts dual roles in marine ecosystems. It exhibits biological activity at trace concentrations but becomes toxic at elevated levels, which are determined by concentration thresholds and organismal sensitivity [5,6]. This trace metal can disrupt neurophysiological function by inhibiting the activity of key enzymes such as acetylcholinesterase, leading to behavioral impairments in marine fauna [7,8]. Additionally, Cd competes with essential metals such as Zn ions at metalloprotein-binding sites, consequently impairing enzymatic processes, inducing oxidative stress cascades, and reducing organismal fitness [9,10]. Furthermore, it engages in complex biogeochemical cycling, and its distribution is closely associated with nitrate and phosphate in the eastern North Pacific regions, suggesting its potential role in nutrient cycling processes [11]. These diverse roles indicate the need for further elucidation of the toxicological effects of this element on marine species.

Sediments function as both sources and sinks of contaminants in marine environments. Thus, these critical biogeochemical interfaces regulate pollutant fluxes across aquatic ecosystems [5,12,13]. Marine sediments can effectively immobilize Cd through adsorption onto reactive substrates such as iron and manganese oxides [14], thereby affecting the bioavailability of Cd to benthic organisms.

Benthic foraminifera are among the most abundant meiofaunal groups in marine sediments. They are characterized by their minute size, high species richness, wide distribution, short life spans, and high environmental sensitivity [15]. Moreover, most foraminiferal species secrete tests composed of calcium carbonate, which are well preserved in coastal environments [16]. Trace elements from seawater, such as Cd, can replace calcium or carbonate ions and be incorporated into the calcitic structure during test formation [17]. These attributes make benthic foraminifera particularly relevant in studies of ancient environments and climate reconstruction, marine pollution monitoring, biogeochemical cycling, and energy flow [18]. For decades, researchers have used benthic foraminifera as bioindicators of heavy metal contamination in marine environments by analyzing the changes in their abundance, community composition, ultrastructure and morphology, and test geochemistry [16,19,20,21,22]. For instance, Qiao et al. [23] identified Cd as a key driver of spatial distribution and compositional dynamics of active foraminiferal assemblages in the Zhejiang coastal waters of the East China Sea.

Traditional assessment of foraminiferal communities relies on stereomicroscopic identification based on test morphology [24,25,26,27]. Although this approach remains the gold standard for taxonomic validation, it is time-consuming, requires specialized expertise, and cannot reliably identify small (<63 μm) or morphologically similar species [25]. Environmental DNA/RNA (eDNA/eRNA) metabarcoding has emerged as a powerful alternative in recent years. It enables high-throughput, sensitive, and environment-friendly assessment of biodiversity [28,29,30,31]. In particular, metabarcoding of eRNA can characterize active cells more effectively, offering a more accurate reflection of their responses to environmental changes [32]. However, metabarcoding has some limitations, including the inability to assess morphological variations in tests and a lack of absolute quantification of foraminiferal abundance [33]. Therefore, integrating stereomicroscopy with metabarcoding is recommended for a comprehensive and ecologically representative assessment of benthic foraminiferal responses to marine environmental changes.

Most studies on the effects of heavy metals on foraminifera have evaluated either morphological deformities or community shifts using a single molecular marker, with a few integrating multiple approaches [22,27,29]. To address this gap, we aimed to integrate morphological analyses with eDNA/eRNA metabarcoding to analyze test geochemistry. Our objectives were to (1) quantify 12 elements in foraminiferal tests and evaluate their relationship with Cd exposure; (2) assess changes in foraminiferal abundance, community composition, and diversity; and (3) identify tolerant versus sensitive taxa.

## 2. Materials and Methods

### 2.1. Sample Collection

Sediment sampling was conducted in Yueqing Bay (121°03′09.03″ E, 28°02′51.65″ N). This region is characterized by low contamination and ecological risk of Cd and a high diversity of active benthic foraminiferal assemblages [34]. Water temperature, salinity, and pH were recorded in situ using a Multiparameter Water Quality Meter (YSI Incorporated, Yellow Springs, OH, USA) to replicate ambient field conditions in laboratory culture experiments. Surface sediment samples were collected from the study area; only the upper layer (~2 cm) was retained, homogenized, and sieved through a 500-μm mesh to remove macrofauna. The processed <500 μm fraction, containing intact benthic microfauna, was immediately transferred into an insulated container and overlaid with site-collected seawater to preserve environmental conditions and minimize physiological stress. Samples were promptly transported to the laboratory.

### 2.2. Culture Experiments and Subsampling

Artificial seawater solutions with six different Cd^2+^ concentrations, and a control, were prepared to assess the effects of Cd on benthic foraminiferal communities. The selected final concentrations of Cd^2+^ solutions were 0 (control), 0.05, 0.2, 1, 3.5, 7, and 70 μg/L, which encompass a complete spectrum ranging from environmental baseline levels to polluted concentrations. The rationale for selecting this concentration gradient is as follows: (1) The concentration range of Cd in routine water samples from Yueqing Bay (2020–2023) was 0.010–0.200 μg/L, with an average value of 0.050 μg/L (unpublished data). Therefore, the concentrations of 0.05 and 0.2 μg/L were set to reflect levels close to the environmental background concentrations. (2) According to the Fishery Water Quality Standard (GB11607-1989 [35]), the permissible limit for Cd is ≤ 5 μg/L. Consequently, the intermediate concentrations of 1 and 3.5 μg/L were selected. (3) The higher concentrations of 7 and 70 μg/L were established based on the Integrated Wastewater Discharge Standard (GB8978-1996 [36]), which stipulates that Cd ≤ 100 μg/L is the permissible limit. The culture temperature was maintained at 24 °C. The salinity and pH of the culture medium were adjusted to 19.7 psu and 7.33, respectively, to replicate the in situ conditions at the sampling points.

The experimental setup consisted of a custom-fabricated culture system (40 cm × 40 cm × 20 cm) containing 25 individual culture chambers (7 cm × 7 cm × 4 cm). Following the gentle siphoning of the overlying seawater, the sediments were homogenized. Three samples were extracted from the homogenized sediments (T0) to establish the baseline characterization of benthic foraminiferal communities, the concentrations of Cd in sediments, and the elemental composition (12 elements) of benthic foraminifera tests. The remaining sediment was distributed into each chamber (20 g per chamber). The culture system (40 cm × 40 cm × 20 cm) was then filled with 16 L of the Cd^2+^ solutions of different concentrations, allowing the sediment in each chamber to be submerged. During the experiment, aeration was maintained using multichannel peristaltic pumps, and cultures were supplemented with live *Nitzschia closterium* twice weekly as a food source.

Sampling was conducted over an 8-week experimental period, with subsampling conducted at six time points: one week (T1), two weeks (T2), three weeks (T3), four weeks (T4), six weeks (T6), and eight weeks (T8). At each time point, one chamber of sediment was used to measure Cd concentrations, another was stained with buffered Rose Bengal dye for morphological analysis of living benthic foraminifera, and one was preserved in labeled 50 mL polypropylene tubes, followed by immediate flash-freezing in liquid nitrogen and subsequent storage at −80 °C for metabarcoding analysis. In particular, one chamber was collected to analyze the concentrations of 12 elements in the benthic foraminiferal tests at T8 (Table S1). Additionally, a 250 mL aliquot of water was collected and filtered through a 0.45 µm filter membrane, immediately acidified with 0.5 mL 80% nitric acid, and refrigerated for the chemical analysis of Cd.

### 2.3. Analysis of Cd Concentration in Water and Sediment

An accurate 40 mL water sample was measured, and the pH was adjusted to 5–6 using 6 mol/L ammonium acetate buffer. Next, an ammonium acetate solution, ammonium pyrrolidine dithiocarbamate, and sodium diethylaminodithiocarbamate were mixed, and a mixed solution of methyl isobutyl ketone and cyclohexane was added to perform liquid–liquid extraction. The upper organic phase was subjected to back-extraction using a mixture of nitric acid and ultrapure water. The back-extracted solution was analyzed to determine Cd concentration in seawater using graphite furnace atomic absorption spectrophotometry (GFAAS; Agilent Technologies Co. Ltd., Santa Clara, CA, USA). Each sample was analyzed in duplicate. A certified standard sample for Cd in seawater (Cd content: 36.3 ± 2.0 µg/L) was used as quality control material to calibrate and ensure the accuracy of water Cd measurements. The detection limit for Cd in water was 0.01 μg/L, as determined using GFAAS.

The sediment samples were freeze-dried in a freeze dryer for approximately 72 h, ground, and then passed through a 160-mesh nylon sieve. Samples (0.2 g) were accurately weighed and microwave-digested with 5 mL nitric acid, 4 mL hydrofluoric acid, and 2 mL perchloric acid in a PTFE digestion tank. After digestion, 4 mL of 1% nitric acid was added, and the solution was diluted to 25 mL with ultrapure water, homogenized by shaking, and centrifuged at 4000 rpm for 5 min. The supernatant was collected for Cd analysis using GFAAS. Each sample was analyzed in duplicate. A certified reference material (Cd content: 0.20 ± 0.04 µg/g) was used for quality assurance. The detection limit for Cd in sediments was 0.04 μg/L, as determined using GFAAS.

### 2.4. Element Analysis in the Tests of Benthic Foraminifera

Eight sediment samples from T0 and T8 were washed over a 63-μm mesh sieve to remove clay materials. Using fine synthetic hairbrushes, intact and clean specimens of benthic foraminifera were manually picked from the sieved sediment under a stereomicroscope. The picked foraminiferal specimens were immediately transferred into ultrapure water and rinsed multiple times to remove any adhering fine sediment particles and salts from shell surfaces. All labware that contacted the sample was made of polypropylene or polyethylene and was pre-cleaned by soaking in 10% trace metal-grade nitric acid for more than 24 h, followed by thorough rinsing with ultrapure water. We concurrently processed procedural blanks to monitor and correct for any potential background contamination introduced during the entire pretreatment process. The pre-treated benthic foraminiferal tests were used to determine the concentrations of 12 elements: Cd, Ca, Mg, Ba, Fe, Mn, Cu, Zn, Pb, Cr, As, and Ni. All selected tests were dried in a drying oven at 60 °C, weighed, and transferred to 10 mL centrifuge tubes. Samples were crushed using glass rods and subjected to ultrasonic cleaning with anhydrous ethanol and ultrapure water to remove clay particles. Subsequently, 10% hydrogen peroxide was added to degrade the organic contaminants. The cleaned samples were dried again in a drying oven at 60 °C and reweighed.

The pretreated test samples (>2 mg) were digested in microwave digestion vessels with a mixture of nitric acid and hydrogen peroxide. After digestion, samples were dried on an acid removal rack and then diluted to 10 mL with ultrapure water in a volumetric flask. Finally, the clear supernatant was analyzed to measure the concentrations of the 12 elements using inductively coupled plasma mass spectrometry (Agilent Technologies Co. Ltd.). Each sample was analyzed in duplicate. The certified reference material for scallop powder was used for quality control of ICP-MS measurements, covering all 12 target elements. The detection limits for Cd, Ca, Mg, Ba, Fe, Mn, Cu, Zn, Pb, Cr, As, and Ni, as determined using ICP-MS, are 0.002, 1, 1, 0.02, 1, 0.1, 0.05, 0.5, 0.02, 0.05, 0.002, and 0.2 μg/g, respectively.

### 2.5. Morphological Analysis

Sediment samples were sieved through a 63-μm mesh to eliminate silt-clay fractions. The retained fractions were stained in Rose Bengal solution for 48 h to differentiate biogenically active foraminiferal specimens (stained) from inactive ones (unstained). Following staining, samples were oven-dried at 60 °C to achieve constant mass. Quantitative enumeration and morphological characterization of the foraminifera were performed using a Leica M205FCA stereomicroscope (Leica, Wetzlar, Germany).

### 2.6. eDNA and eRNA Metabarcoding

#### 2.6.1. eDNA and eRNA Extraction

eDNA and eRNA were extracted from sediment samples using the FastDNA^®^ SPIN Kit for Soil and FastRNA^®^ Pro Soil Direct Kit (both MP Biomedicals, Irvine, CA, USA), respectively, following the manufacturers’ protocols. All extractions included negative controls to monitor cross-contamination. Nucleic acid purity and concentration were assessed using a NanoDrop 2000 spectrophotometer (Thermo Fisher Scientific, Waltham, MA, USA), and the integrity was verified using 1% agarose gel electrophoresis. The extracted eRNA was purified using a Turbo DNA-free kit (Thermo Fisher Scientific) and subsequently reverse-transcribed to cDNA using a High-Capacity cDNA Reverse Transcription Kit (Thermo Fisher Scientific). The eDNA, eRNA, and cDNA were cryopreserved at −80 °C until subsequent experiments.

#### 2.6.2. PCR Amplification and Sequencing

The small subunit ribosomal DNA region was amplified using the foraminiferal-specific primers s14F3 (5′-ACGCAMGTGTGAAACTTG-3′) and s17 (5′-CGGTCACGTTCGTTGC-3′) [37,38]. PCR (total volume of 20 μL) was performed in triplicate on both eDNA and cDNA templates. Samples contained eDNA or cDNA templates, 10 μL of 2 × Pro Taq, and 0.8 μL of each primer (5 μM), and PCR was conducted under the following optimized thermal cycling conditions: initial denaturation at 94 °C for 1.5 min; 25 cycles at 94 °C for 60 s, 55 °C for 60 s, and 72 °C for 45 s; followed by additional 10 cycles at 94 °C for 30 s and 55 °C for 30 s; and extension at 72 °C for 2 min. Negative controls were included for each experiment.

PCR products from triplicate samples were pooled and then electrophoresed on 1.5% agarose gels. Target bands were subsequently excised for purification using the AxyPrep DNA Gel Extraction Kit (Axygen Biosciences, Union City, CA, USA). Purified PCR products were quantified using a NanoDrop 2000 spectrophotometer (Thermo Fisher Scientific). Equimolar amplicons were pooled and paired-end sequenced on an Illumina MiSeq PE300 platform (Illumina, San Diego, CA, USA) by Majorbio Bio-Pharm Technology Co., Ltd. (Shanghai, China). Raw FASTQ files were deposited in the NCBI Sequence Read Archive (https://www.ncbi.nlm.nih.gov/sra, accessed on 23 April 2025) under BioProject accession number PRJNA1254124.

#### 2.6.3. Bioinformatic Analysis

Raw paired-end Illumina sequences were merged using FLASH (version 1.2.7) [39]. High-quality reads were filtered, and chimeric sequences were detected and removed using the QIIME pipeline (version 1.9.1) [40]. The filtered sequences were clustered into operational taxonomic units (OTUs) at a 97% similarity threshold using the UPARSE pipeline (version 7.0.1090) [41]. Representative OTU sequences were aligned against the NCBI nucleotide (nt) database using BLAST (https://blast.ncbi.nlm.nih.gov/Blast.cgi, accessed on 22 February 2025). OTUs not assigned to foraminifera at the phylum level were excluded from downstream analyses. To address the sequencing depth heterogeneity, foraminiferal eDNA and eRNA datasets were rarefied to 26,184 sequences per sample to ensure equitable community comparisons.

### 2.7. Statistical Analysis

All statistical analyses were conducted using SPSS (version 20; IBM Corp., Armonk, NY, USA). An independent samples t-test was used along with Pearson correlation analysis for normally distributed data; a nonparametric test and Spearman correlation analysis were used for non-normally distributed data. Statistical significance was set at *p* < 0.05. The Chao1 and Shannon indices were calculated using Mothur (version 1.30.2). Benthic foraminiferal communities with relative abundances >10% were defined as dominant taxa [34]. Hierarchical clustering was performed using the average linkage method based on Bray–Curtis dissimilarity.

## 3. Results

### 3.1. Concentrations of Cd in Water and Sediments

**Figure 1:**
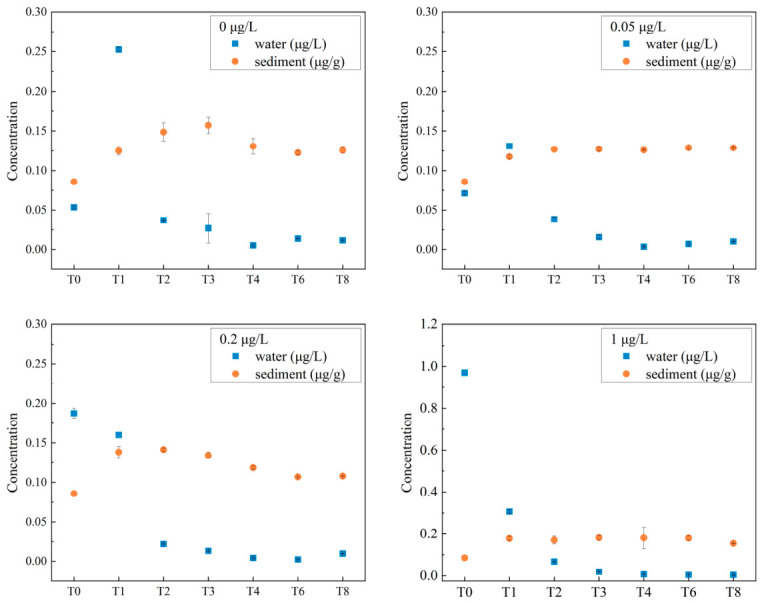

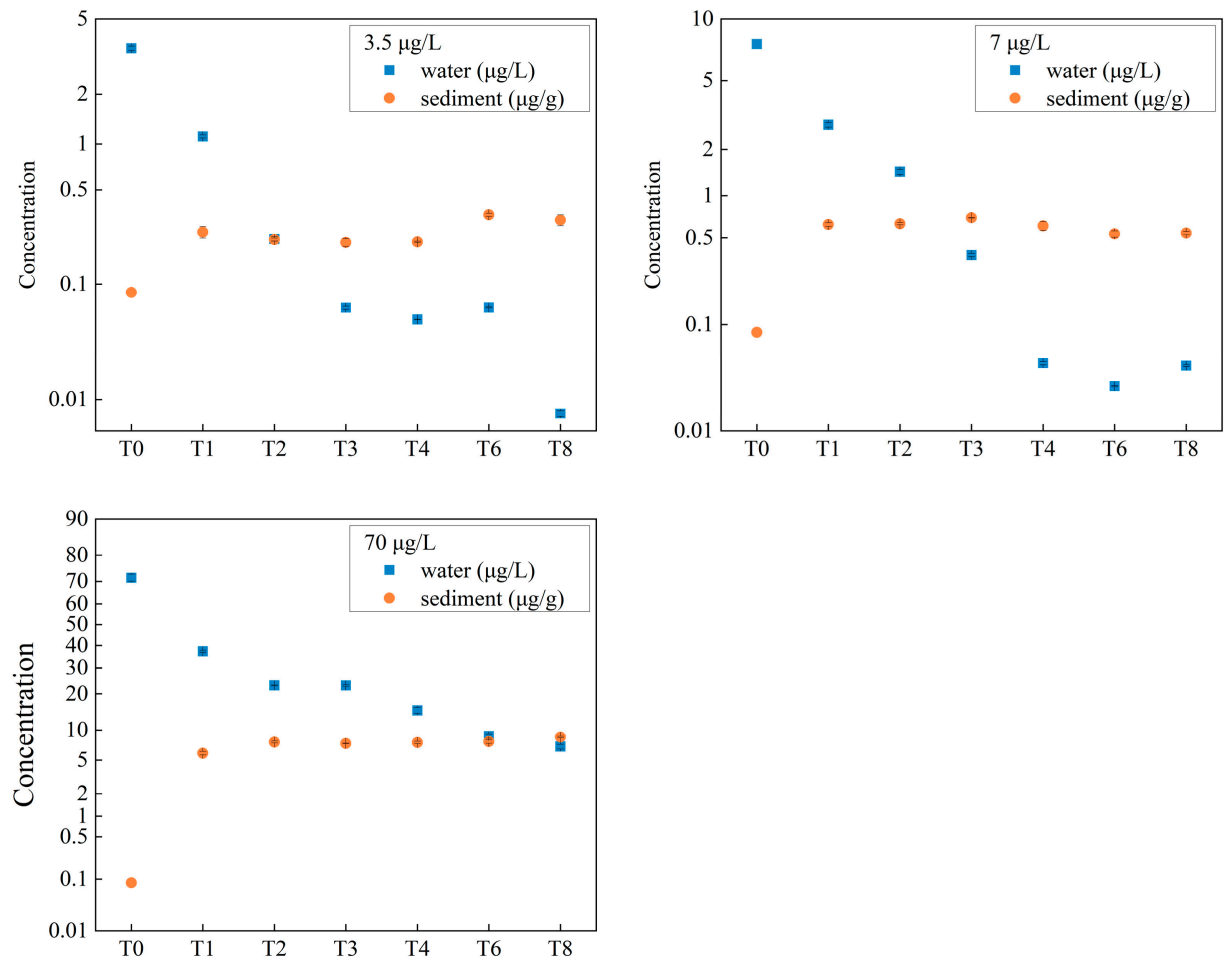
Trends in Cd concentrations in water and sediment samples over time. T0–T8 represent sample collection time points in weeks. T0, week 0; T1, week 1; T2, week 2; T3, week 3; T4, week 4; T6, week 6; T8, week 8.

Temporal variations in Cd concentrations in both water and sediments are shown in Figure 1. Cd concentrations in water tended to decrease with an increase in culture time. A significant increase in sediment Cd concentrations was observed in the first week (T1) of the experiment, after which the concentrations remained relatively stable. The Cd concentration in sediments increased with increasing Cd concentration in culture solutions. Statistical comparisons between each treatment group and the control group were performed using either the independent-samples t-test (for the 0.2 and 3.5 μg/L groups) or the Mann–Whitney U test (for the 0.05, 1, 7, and 70 μg/L groups), depending on the normality of data. No significant differences in sediment Cd concentration were observed between the control group and the low concentration groups (0.05 and 0.2 μg/L; *p* > 0.05). Conversely, sediment Cd concentrations in the 1, 3.5, 7, and 70 μg/L groups were significantly higher than those in the control group (*p* < 0.05 for each comparison).

### 3.2. Concentrations of 12 Elements in the Tests of Benthic Foraminifera

**Figure 2:**
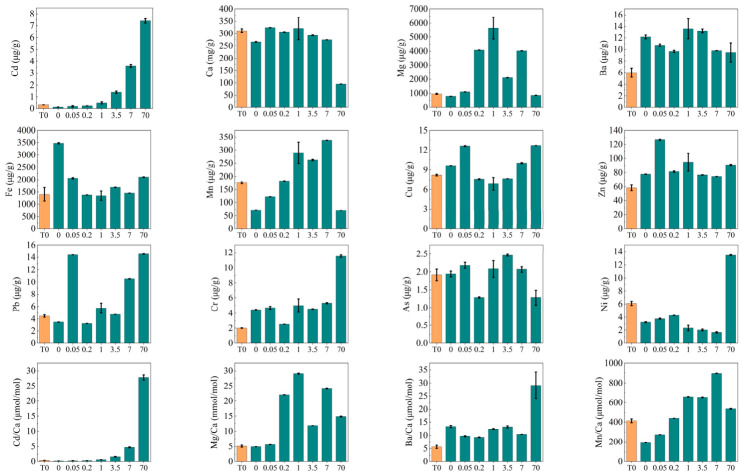
Concentrations of the 12 elements in the tests of benthic foraminifera at T0 and T8. The first value on the x-axes (leftmost) represents T0; all other values on the x-axes represent T8 under different concentrations.

The concentrations of the 12 elements in the benthic foraminiferal tests at T0 and T8 are shown in Figure 2. Both Cd concentration and the Cd/Ca ratio increased with an increase in Cd concentration in the culture solutions. The concentration of Ca in the benthic foraminiferal tests at the Cd concentration of 70 μg/L was lower than that at other concentrations and at T0. The concentrations of Mg and As and the Mg/Ca ratio in the benthic foraminiferal tests initially increased with Cd exposure but decreased at higher Cd concentrations in the culture solution. The concentrations of Ba, Zn, and Cr and the Ba/Ca ratio in the benthic foraminiferal tests were lower at T0 than at T8; among these, Cr and the Ba/Ca ratio increased at the Cd concentration of 70 μg/L. The concentrations of Fe and Cu in the benthic foraminiferal tests first decreased and then increased with increasing Cd concentrations in the culture solution. The concentration of Mn and the Mn/Ca ratio in the benthic foraminiferal tests increased with an increase in Cd concentration in the culture solution, except at the Cd concentration of 70 μg/L, where both decreased. The concentration of Pb in the benthic foraminifera increased with increasing Cd concentrations in the culture solution, except at a Cd concentration of 0.05 μg/L. Conversely, the concentration of Ni in the benthic foraminiferal tests decreased with increasing Cd concentration in the culture solution, except at the Cd concentration of 70 μg/L, where an increase was observed.

Spearman rank correlation analysis showed that the Cd concentration of the tests had a significantly positive relationship with the Cd concentration in the sediment (*p* < 0.05). It was also significantly positively correlated with the Cd/Ca and Mn/Ca ratios (*p* < 0.05).

### 3.3. Benthic Foraminiferal Absolute Abundance

**Figure 3:**
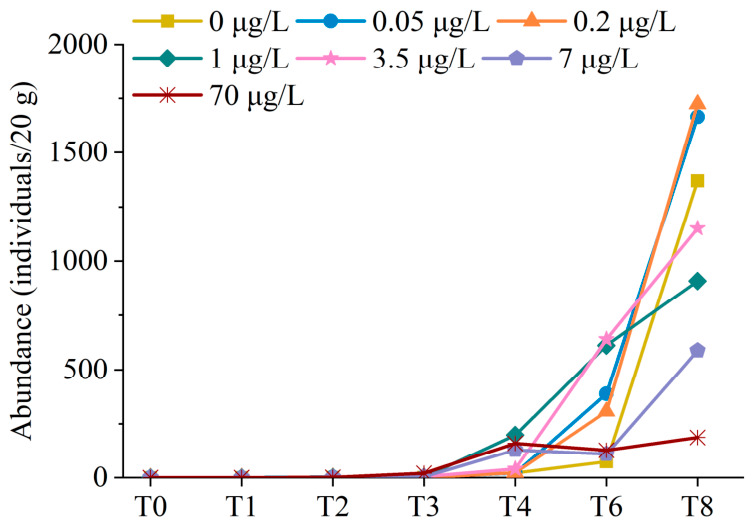
Absolute abundance of living benthic foraminifera in sediments. T0–T8 represent sample collection time points in weeks. T0, week 0; T1, week 1; T2, week 2; T3, week 3; T4, week 4; T6, week 6; T8, week 8.

A total of 10,501 specimens of living (Rose Bengal-stained) benthic foraminifera were observed using a stereomicroscope (Figure 3). Hyaline foraminifera were the most abundant, accounting for approximately 90%, followed by porcelaneous (9.28%) and agglutinated foraminifera (0.72%). No significant changes in foraminiferal abundance were observed during the first three weeks. However, the absolute abundance of live foraminifera generally increased after the third week (T3). At the eighth week of culture (T8), the absolute abundance of living foraminifera in low Cd treatment groups (0.05 and 0.2 μg/L) was higher than that in the control group. However, the absolute abundance of living foraminifera in the high-Cd treatment groups (1, 3.5, 7, and 70 μg/L) was lower than that in the control group.

### 3.4. Benthic Foraminiferal Community Diversity, as Assessed Using eDNA and eRNA Metabarcoding

**Figure 4:**
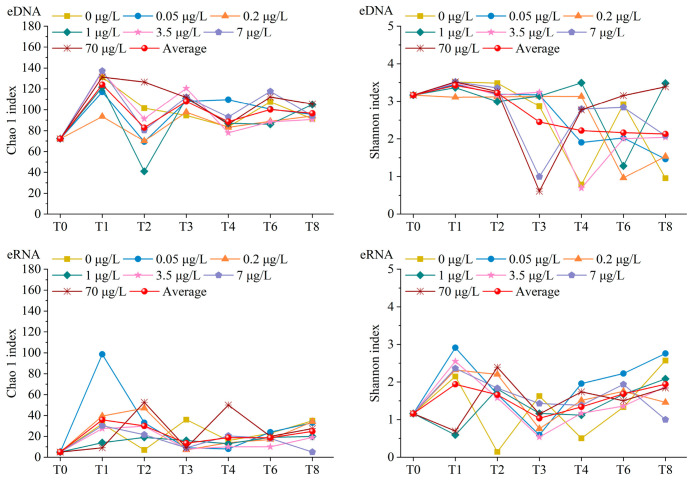
Trends in the Chao1 and Shannon indices of benthic foraminiferal communities at the OTU level, as assessed using eDNA and eRNA metabarcoding. T0–T8 represent sampling time points. T0, week 0; T1, week 1; T2, week 2; T3, week 3; T4, week 4; T6, week 6; T8, week 8.

The richness and diversity of the benthic foraminiferal communities were estimated using the Chao1 and Shannon indices, respectively (Figure 4). At the eDNA level, both indices declined over time and were lower in the low concentration groups than in the high concentration groups. Conversely, eRNA-based indices of benthic foraminiferal communities exhibited an initial decline and then increased over time; higher values were observed in the low concentration groups than in the high concentration groups. Overall, both the richness and diversity revealed by eDNA were higher than those revealed by eRNA.

### 3.5. Benthic Foraminiferal Community Composition, as Assessed Using eDNA and eRNA Metabarcoding

**Figure 5:**
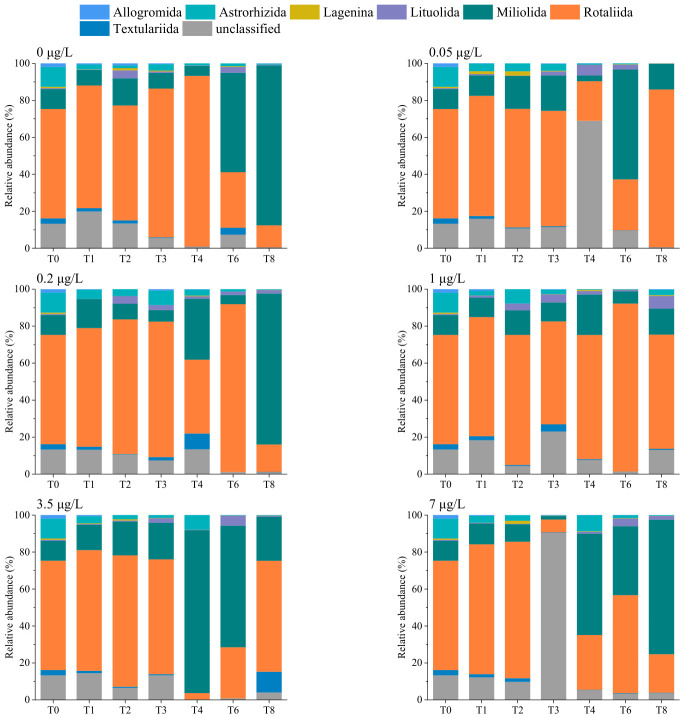

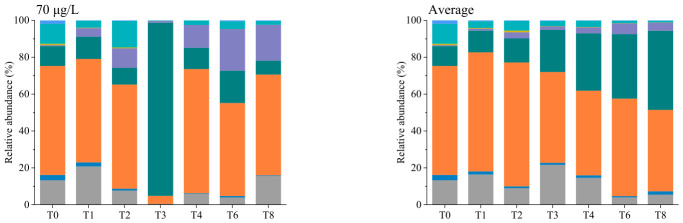
Benthic foraminiferal community composition within sediments at the order level revealed by eDNA metabarcoding. T0–T8 represent sampling time points. T0, week 0; T1, week 1; T2, week 2; T3, week 3; T4, week 4; T6, week 6; T8, week 8.

Benthic foraminiferal community composition at the order level, as revealed by eDNA metabarcoding, is shown in Figure 5. Seven orders were identified—Allogromida, Astrorhizida, Lagenina, Lituolida, Miliolida, Rotaliida, and Textulariida. Among these, Rotaliida was the most abundant order, followed by Miliolida. The relative abundances of Rotaliida and Astrorhizida gradually decreased with an increase in culture time, whereas those of Miliolida and Lituolida increased (Table S2). ANOVA with Tukey–Kramer post hoc test indicated that Lituolida exhibited significant differences among groups (*p* = 0.002; Table S3), with a higher relative abundance at the 70 μg/L Cd concentration than in other groups.

**Figure 6:**
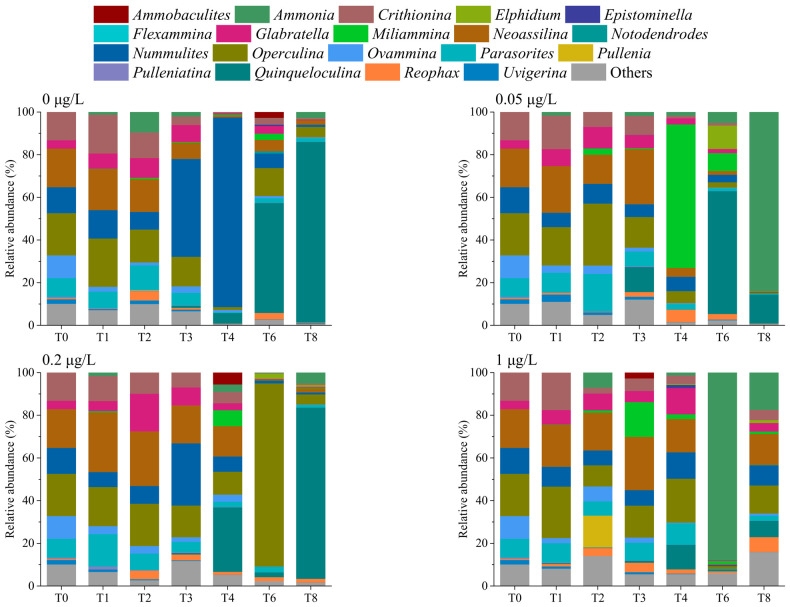

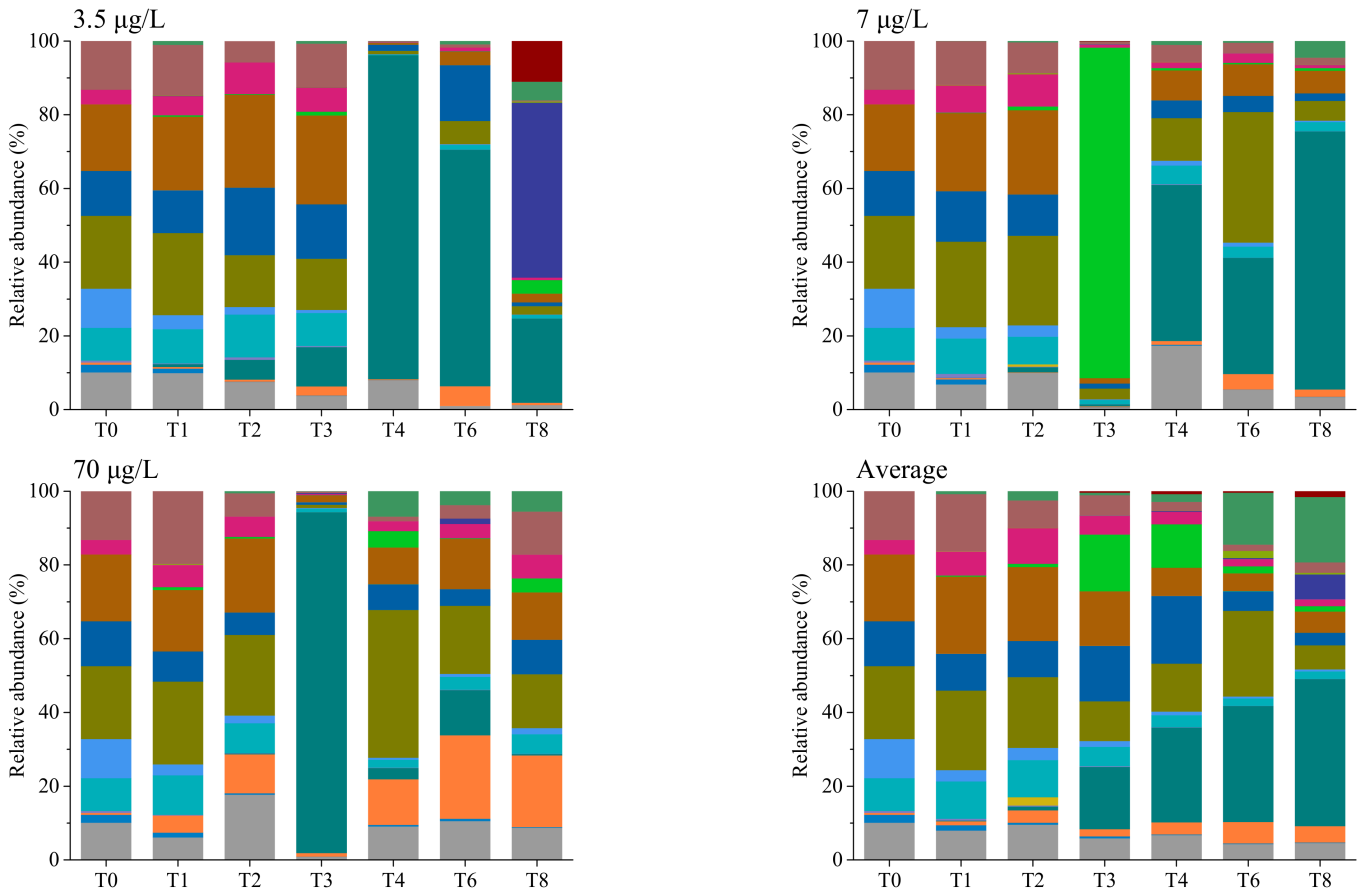
Benthic foraminiferal community composition within sediments at the genus level, as revealed by eDNA metabarcoding. T0–8 represent sampling time points. T0, week 0; T1, week 1; T2, week 2; T3, week 3; T4, week 4; T6, week 6; T8, week 8.

In total, 72 taxa were identified at the genus level using eDNA metabarcoding (Figure 6, Table S4). The genus *Quinqueloculina* exhibited the highest average relative abundance, followed by *Operculina*, *Neoassilina*, and *Nummulites*. The community composition of benthic foraminifera remained relatively stable during the first three weeks, with *Neoassilina*, *Operculina,* and *Nummulites* being the dominant taxa. At T4 and T5, *Quinqueloculina* became dominant, alongside *Operculina* and *Nummulites.* Furthermore, the dominance of *Quinqueloculina* and *Ammonia* increased at T8. ANOVA with Tukey–Kramer post hoc test indicated that *Reophax* showed significant differences among groups (*p* = 0.000; Table S3), with its relative abundance peaking in the 70 μg/L Cd group.

**Figure 7:**
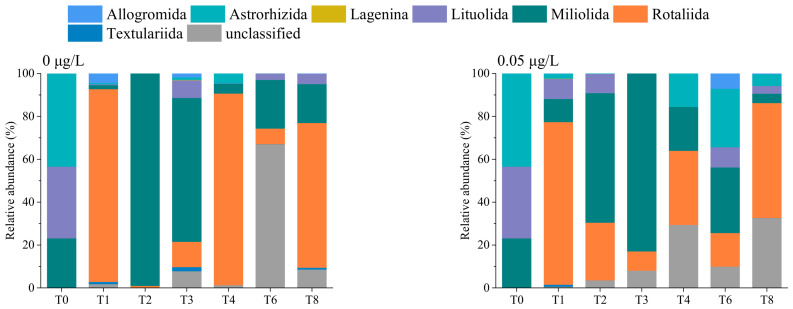

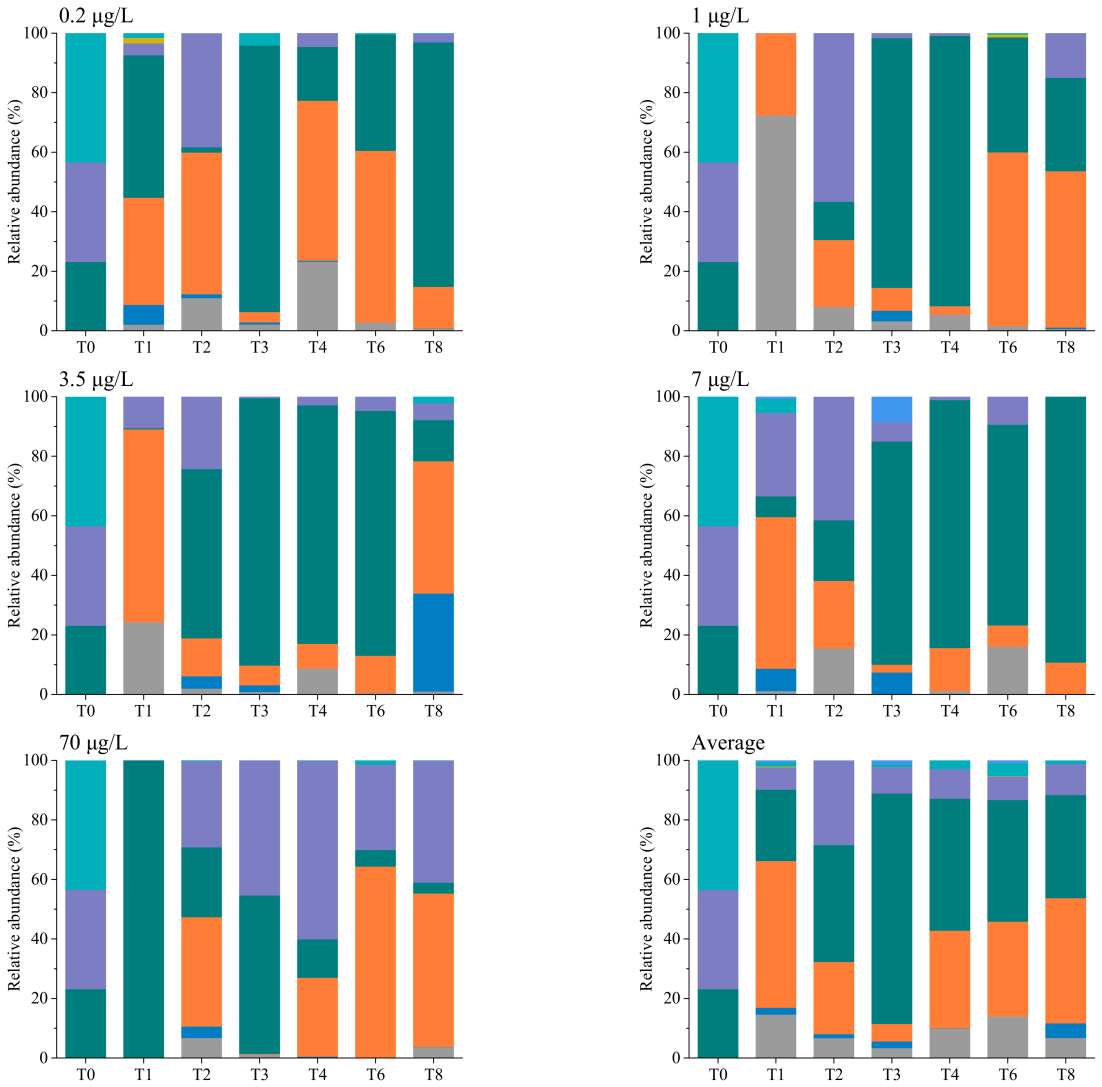
Benthic foraminiferal community composition within sediments at the order level, as determined using eRNA metabarcoding. T0–T8 represent sampling time points. T0, week 0; T1, week 1; T2, week 2; T3, week 3; T4, week 4; T6, week 6; T8, week 8.

Figure 7 shows the benthic foraminiferal community composition at the order level, as revealed by eRNA metabarcoding. Seven orders were identified: Allogromida, Astrorhizida, Lagenina, Lituolida, Miliolida, Rotaliida, and Textulariida. Among these, Miliolida was the most abundant, followed by Rotaliida and Lituolida. The relative abundance of Miliolida initially increased and then decreased over time, whereas that of Rotaliida showed the opposite trend, that is, it first decreased and then increased (Table S5). ANOVA with Tukey–Kramer post hoc test indicated that the relative abundance of Lituolida was significantly higher at 70 μg/L Cd concentration than at 0.05 μg/L and in the control group (*p* = 0.038; Table S3).

**Figure 8:**
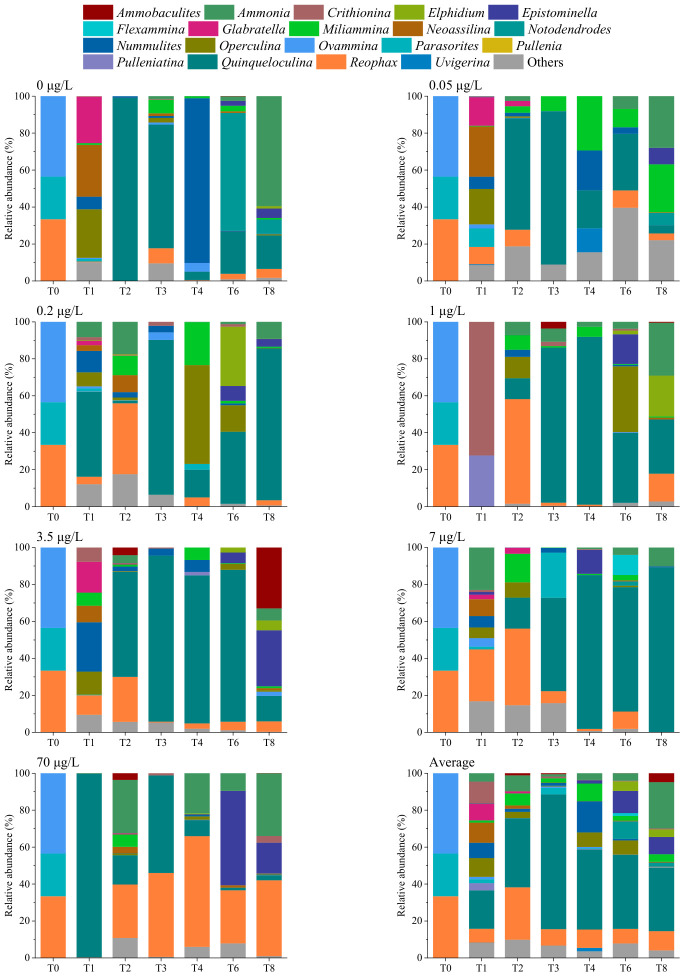
Benthic foraminiferal community composition within sediments at the genus level, as determined using eRNA metabarcoding. T0–T8 represent sampling time points. T0, week 0; T1, week 1; T2, week 2; T3, week 3; T4, week 4; T6, week 6; T8, week 8.

In total, 57 taxa were identified at the genus level using eRNA metabarcoding (Figure 8, Table S6). *Quinqueloculina* exhibited the highest average relative abundance, followed by *Reophax* and *Ammonia*. The relative abundances of *Quinqueloculina* and *Reophax* initially increased and then decreased over time. *Quinqueloculina* and *Reophax* had the highest relative abundances at T3 and T2, respectively. Furthermore, the relative abundances of *Ammonia*, *Elphidium,* and *Epistominella* increased at T8. The average relative abundances of *Reophax* and *Epistominella* showed an overall upward trend, whereas those of *Ovammina*, *Notodendrodes*, *Miliammina*, *Nummulites,* and *Neoassilina* declined with an increase in Cd concentration in the culture solution. ANOVA with Tukey–Kramer post hoc test indicated that *Reophax* showed significant differences among groups (*p* = 0.030; Table S3). Moreover, its relative abundance in the 70 μg/L Cd group was significantly higher than that in other groups, especially after T4.

### 3.6. Relationships Between Benthic Foraminifera and Cd Concentrations

**Figure 9:**
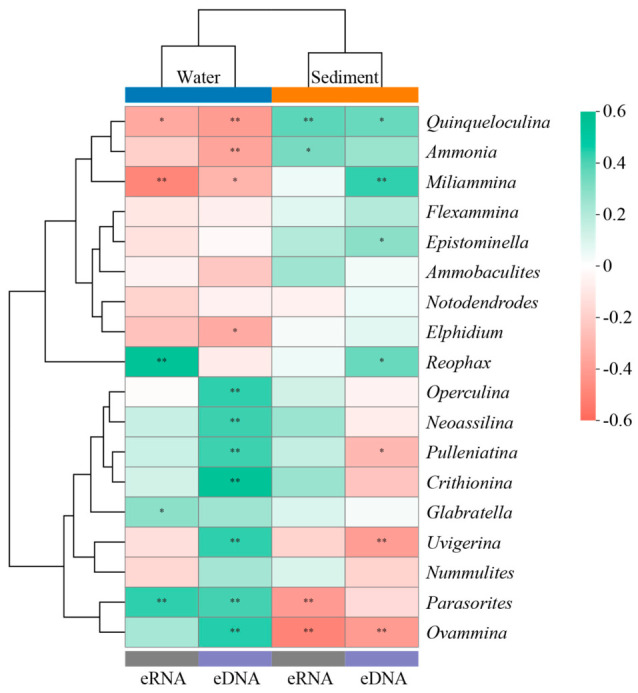
Heatmaps indicating the correlations between the benthic foraminiferal community at the genus level and Cd concentrations. Hierarchical clustering was performed using the average linkage method based on Bray–Curtis dissimilarity. * indicates 0.01 ≤ *p* < 0.05; ** indicates *p* < 0.01.

To clarify the relationship between Cd levels and the benthic foraminiferal community, the dominant genera were selected for Spearman’s rank correlation analysis (Figure 9). *Quinqueloculina*, *Ammonia*, and *Miliammina* were positively correlated with Cd concentrations in sediments and negatively correlated with Cd concentrations in water (*p* < 0.05). In contrast, *Parasorites* and *Ovammina* exhibited negative correlations with Cd concentrations in sediments and positive correlations with Cd concentrations in water (*p* < 0.05).

## 4. Discussion

### 4.1. Cd Concentrations in Water and Sediments

Heavy metals are among the primary pollutants in aquatic ecosystems. In this study, the enrichment of Cd in the sediments likely contributed to the decrease in Cd concentration in water (Figure 1). The adsorption–desorption behavior of heavy metals is strongly influenced by the physicochemical characteristics of the surrounding water and sediments [30,31,32,33,34,37,38,39,40,41,42,43,44]. Furthermore, the presence of organic matter can increase the ability of sediments to sequester Cd [45].

The concentration of Cd in sediments increased with the concentration of Cd in culture solutions (Figure 1). Sedimentary Cd concentrations in the low concentration groups did not significantly differ from those in the control group, which is consistent with the results of a previous field investigation in Yueqing Bay [46]. Conversely, Cd concentrations in the sediments at high concentrations were significantly higher than those in the control group (*p* < 0.05). The concentrations of Cd in sediments in the 70 μg/L group ranged from 5.92 μg/g to 8.61 μg/g. These concentrations exceed the effect range low value for Cd (1.2 μg/g) outlined in the sediment guidelines established by the US Environmental Protection Agency [47].

### 4.2. Effects of Cd Pollution on the Abundance of Benthic Foraminifera

Cd affected the growth rate and abundance of benthic foraminifera in this study, which is consistent with the results of previous studies [48,49]. A gradual increase in Cd concentration primarily impaired normal growth and induced morphological abnormalities in *Pararotalia nipponica*; concentrations >2 μg/L led to mortality in more than half of the individuals [48]. In the present study, higher Cd concentrations in the culture solution correlated with lower absolute abundance of live foraminifera (Figure 3). However, the absolute abundance of live foraminifera showed an overall increasing trend after T3, even at high Cd concentrations (Figure 3). This observation indicates the tolerance of some foraminiferal species to Cd. For example, a relatively high concentration of Cd (33 μg/L) did not result in *Astrammina rara* mortality in a previous study [50]. Similarly, a study on the effects of Cd, Pb, and Zn on benthic foraminiferal assemblages [51] revealed that Cd had the least acute effect on total abundance, even at the highest concentrations. Brouillette [52] found no marked differences in *Psammophaga simplora* abundance at Cd concentrations up to 3.6 ppm and in the controls. However, an acute response was observed when Cd concentration increased to 10.36 ppm. The morphological data presented in the present study were derived from single measurements per treatment per time point. Consequently, no error bars can be calculated for these data. Future studies should incorporate true biological replicates (e.g., multiple chambers per treatment per time point) to enable accurate quantification of variability and more robust statistical inference.

### 4.3. Effects of Cd Pollution on the Community Structure and Diversity of Benthic Foraminifera

Previous studies reported a decline in benthic foraminiferal diversity upon exposure to heavy metals [27,51]. Liu et al. [53] reported significantly negative correlations between Cd concentrations and foraminiferal diversity indices in Bohai Bay. In this study, the Chao1 and Shannon indices were lower in the low-Cd concentration groups than in the high-Cd concentration groups at the eDNA level. Conversely, these biodiversity indices demonstrated an inverse pattern at the eRNA level; significantly higher values were observed in the low-Cd concentration groups than in the high-Cd concentration groups (Figure 4). eDNA can originate from living, dormant, and even dead organisms. Therefore, it represents a cumulative record of recent community presence. In contrast, eRNA is rapidly degraded after cell death and reflects only the metabolically active fraction [23]. Consequently, eDNA tends to integrate the past community and may exhibit a delayed or dampened response to environmental stress, whereas eRNA provides a real-time overview of the active assemblage responding to current conditions [26,52]. These differences explain the opposite temporal trends observed between eDNA- and eRNA-derived diversity indices. These opposite patterns further demonstrate that RNA-based diversity better reflects environmental changes, which is consistent with the results of previous studies [32,54,55]. However, eRNA methodology cannot fully substitute for the eDNA technology, owing to the susceptibility of eRNA to degradation under environmental influences. This susceptibility can result in the loss of crucial biological information. Furthermore, eRNA methodologies have more intricate and demanding requirements for sample collection and processing than do eDNA techniques [38,56]. Therefore, these two complementary approaches should be strategically integrated to leverage their respective advantages. This approach can effectively minimize analytical bias and enhance detection accuracy through synergistic applications [54,57].

Benthic foraminifera exhibit differential responses to Cd pollutants based on their species susceptibility. In a previous study, the rotaliids *Haynesina germanica* and *Ammonia tepida* were tolerant to high concentrations of Cd, whereas the monothalamids *Ovammina opaca* and *Psammophaga simplora* were highly sensitive to Cd exposure [51]. Similarly, the intensity of the deformation of the coastal benthic foraminifera *Pararotalia nipponica* increased with increasing Cd concentrations, indicating its sensitivity to Cd [48]. In the present study, *Quinqueloculina*, *Ammonia*, and *Miliammina* showed tolerance to Cd pollution, whereas *Parasorites* and *Ovammina* exhibited sensitivity (Figure 9). This finding aligns with the results of previous studies showing that Cd can reduce the rates of oxygen consumption and pseudopodal activity in benthic foraminifera [50,58], thereby affecting the growth and reproduction of sensitive species.

Benthic foraminifera can survive and reproduce at high concentrations of Cd because of the bioavailability of this element and the defense mechanisms of foraminifera. First, Cd is adsorbed by sediments, thereby reducing its bioavailability [59,60]. However, the absence of bioavailability measurements is a limitation of this study. Second, the bioavailability of Cd is affected by environmental conditions. The bioavailability of Cd is reduced at pH > 6.5 [61]. In this study, the pH of the culture medium was adjusted to 7.33. However, the chemical speciation was not determined, which limits interpretation. Therefore, further research exploring its effects on foraminiferal toxicity is warranted. In addition, benthic foraminifera may have developed defense mechanisms against heavy metal-induced stress, including thickening of the inner organic lining and lipid accumulation [29].

Metabarcoding relies on reference databases for taxonomic assignment, and the incomplete coverage of foraminiferal sequences in public repositories remains a major challenge [62]. In this study, only OTUs assigned to the phylum Foraminifera were retained; all unassigned OTUs and those assigned to other phyla were excluded from downstream analyses of diversity and community composition. This approach reduces the risk of false positives. However, it may also discard some genuine foraminiferal sequences that lack close matches in the current database. Therefore, future studies should prioritize the development and curation of comprehensive reference databases for benthic foraminifera, which will, in turn, enable more accurate and reliable metabarcoding assessments of foraminiferal communities.

### 4.4. Effects of Cd Pollution on Elemental Composition of the Foraminiferal Tests

In addition to the abundance, community structure, and diversity of benthic foraminifera, heavy metal pollution can also affect the elemental composition of their tests [27]. As a non-essential element for organisms, Cd does not participate in physiological processes. Therefore, it is considered highly toxic [63]. Foraminifera may incorporate non-essential metals into their tests instead of keeping them in their cells when cellular exclusion mechanisms fail [64,65]. This is a common detoxification mechanism in organisms [66,67].

In this study, Cd concentrations in the tests increased with increasing Cd concentrations in culture solutions (Figure 2), which indicated that Cd was enriched in the foraminiferal tests. This finding is consistent with a previous report that foraminifera can assimilate heavy metals and incorporate them into their calcium carbonate tests during calcification [68,69,70,71]. Sagar et al. [72] conducted culture experiments to quantify the uptake of Mn, Ni, and Cd by live juvenile specimens of *Amphisorus hemprichii*. Changes in the concentrations of Mn, Ni, and Cd in *A. hemprichii* specimens were directly proportional to those in the culture solution at varying concentrations [72]. However, differences were observed in the tendency of different foraminifera to incorporate heavy metals. For example, the rotaliid species *Ammonia tepida* and *Haynesina germanica* incorporated more Cd as its concentration in the water increased, whereas the miliolid species *Quinqueloculina sabulosa* and *Triloculina oblonga* incorporated more Zn and Ni than Cd [73]. Another limitation of the present study is that species-specific metal content was not measured. Therefore, future research should focus on measuring Cd in the tests of hand-picked dominant species to elucidate the enrichment effects of different species on heavy metals.

The increasing Cd concentration in foraminiferal tests with rising Cd exposure (Figure 2) can be explained by the entry of Cd^2+^ into cells via Ca^2+^ channels or Ca^2+^-ATPases, which compete with Ca^2+^ for binding sites and co-precipitate into the calcite test. Cations are absorbed into inorganic calcite by substitution with Ca^2+^ [74], especially when the effective ionic radius of these ions is comparable to that of Ca^2+^. The effective ionic radius of Cd^2+^ (0.95 Å) is comparable to that of Ca^2+^ (1.0 Å), facilitating the incorporation of Cd into calcite. In the present study, Ca concentration in the tests at 70 μg/L Cd was lower than that at other concentrations and at T0 (Figure 2). In addition to Ca, Cd exposure also reduced Mn concentrations in the tests at 70 μg/L Cd concentration (Figure 2). Mn is an essential trace element involved in cellular antioxidant defense. Cd exposure alters Mn content [75]. Both metals are divalent cations transported via common membrane transporters. Thus, Cd may compete with Mn for shared uptake pathways, thereby restricting Mn absorption in Cd-polluted environments [76]. Mn is also a key cofactor for superoxide dismutase (SOD). Therefore, it plays a crucial role in the normal functioning of Mn–SOD. Cd-induced Mn deficiency directly impairs Mn–SOD expression and activity, which, in turn, diminishes the cellular capacity to counteract oxidative stress [77]. Ca concentrations decreased more steeply than Mn concentrations, resulting in an overall increase in the Mn/Ca ratio.

The chemical composition of calcareous foraminifera reflects the physical and chemical environment in which calcification occurs, providing valuable proxies for the study of ancient climates and global changes [78]. For example, foraminiferal Mg/Ca ratios are widely used to reconstruct sea temperatures [79], whereas the Ba/Ca ratio in foraminiferal tests may serve as a useful indicator of the timing and processes associated with deglaciation [80]. The Mn/Ca ratio was recently used to reflect and reconstruct the redox environment of oceanic bottom water and the interstitial water of sediments [81,82]. The Cd/Ca ratios are a well-established proxy for seawater paleonutrient concentrations [83]. In the present study, both Cd/Ca and Mn/Ca ratios were significantly positively correlated with the concentration of Cd (*p* < 0.05), indicating that Cd pollution affected the chemical composition of benthic foraminifera. Therefore, the impact of environmental pollution should be considered when establishing and applying trace element proxies.

## 5. Conclusions

In this study, we explored the effects of varying Cd concentrations on benthic foraminiferal communities using morphological and metabarcoding analyses under controlled laboratory conditions. Higher Cd concentrations in the culture solutions correlated with higher Cd concentrations in the sediments. Exposure to high concentrations of Cd can lead to a decrease in foraminiferal abundance. In total, 72 genera were identified via eDNA metabarcoding, with *Quinqueloculina*, *Operculina*, *Neoassilina,* and *Nummulites* being predominant. Contrastingly, 57 genera were identified by eRNA metabarcoding. Of these, *Quinqueloculina*, *Reophax,* and *Ammonia* were dominant. *Quinqueloculina*, *Ammonia,* and *Miliammina* showed tolerance to Cd, whereas *Parasorites* and *Ovammina* were sensitive to this trace metal. Our findings indicate that Cd was enriched in the foraminiferal tests and that Cd pollution affected the chemical composition of benthic foraminifera. Future studies should employ advanced omics technology, such as genomics, transcriptomics, proteomics, and other approaches, to investigate the genetic mechanisms underlying the response of benthic foraminifera to heavy metal pollution.

## Data Availability

The original contributions presented in this study are included in the article/Supplementary Materials. Further inquiries can be directed to the corresponding author.

## Supplementary Materials

The following supporting information can be downloaded at: https://www.mdpi.com/article/10.3390/microorganisms14051122/s1, Table S1: Experimental design matrix showing the number and usage of culture chambers per Cd concentration per time point; Table S2: Relative abundance (%) of benthic foraminiferal orders over time and under different Cd concentrations, based on eDNA metabarcoding; Table S3: Statistical results (eta-squared and *p*-values) for foraminiferal orders and genera based on DNA and RNA datasets. Significant *p*-values (<0.05) are shown in bold.; Table S4: Relative abundance (%) of benthic foraminiferal genera over time and under different Cd concentrations, based on eDNA metabarcoding; Table S5: Relative abundance (%) of benthic foraminiferal orders over time and under different Cd concentrations, based on eRNA metabarcoding; Table S6: Relative abundance (%) of benthic foraminiferal genera over time and under different Cd concentrations, based on eRNA metabarcoding.
