# Supplementary material for: Response of Benthic Foraminifera to Cadmium Pollution Assessed via Morphological and Metabarcoding Analyses
Source: Microorganisms. 2026 May 15;14(5):1122. doi: 10.3390/microorganisms14051122 (PMC13209411; doi:10.3390/microorganisms14051122)
Supplement: Supplementary file 1 [file microorganisms-14-01122-s001.zip › microorganisms-4236871-supplementary.pdf]

Table S1. Experimental design matrix showing the number and usage of culture chambers per Cd concentration per time point

Time	0 μg/L	0.05 μg/L	0.2 μg/L	1 μg/L	3.5 μg/L	7 μg/L	70 μg/L
T0	Cd<br>Mor<br>Met<br>Ele	/	/	/	/	/	/
T1	Cd (1)<br>Mor (1)<br>Met (1)	Cd (1)<br>Mor (1)<br>Met (1)	Cd (1)<br>Mor (1)<br>Met (1)	Cd (1)<br>Mor (1)<br>Met (1)	Cd (1)<br>Mor (1)<br>Met (1)	Cd (1)<br>Mor (1)<br>Met (1)	Cd (1)<br>Mor (1)<br>Met (1)
T2	Cd (1)<br>Mor (1)<br>Met (1)	Cd (1)<br>Mor (1)<br>Met (1)	Cd (1)<br>Mor (1)<br>Met (1)	Cd (1)<br>Mor (1)<br>Met (1)	Cd (1)<br>Mor (1)<br>Met (1)	Cd (1)<br>Mor (1)<br>Met (1)	Cd (1)<br>Mor (1)<br>Met (1)
T3	Cd (1)<br>Mor (1)<br>Met (1)	Cd (1)<br>Mor (1)<br>Met (1)	Cd (1)<br>Mor (1)<br>Met (1)	Cd (1)<br>Mor (1)<br>Met (1)	Cd (1)<br>Mor (1)<br>Met (1)	Cd (1)<br>Mor (1)<br>Met (1)	Cd (1)<br>Mor (1)<br>Met (1)
T4	Cd (1)<br>Mor (1)<br>Met (1)	Cd (1)<br>Mor (1)<br>Met (1)	Cd (1)<br>Mor (1)<br>Met (1)	Cd (1)<br>Mor (1)<br>Met (1)	Cd (1)<br>Mor (1)<br>Met (1)	Cd (1)<br>Mor (1)<br>Met (1)	Cd (1)<br>Mor (1)<br>Met (1)
T6	Cd (1)<br>Mor (1)<br>Met (1)	Cd (1)<br>Mor (1)<br>Met (1)	Cd (1)<br>Mor (1)<br>Met (1)	Cd (1)<br>Mor (1)<br>Met (1)	Cd (1)<br>Mor (1)<br>Met (1)	Cd (1)<br>Mor (1)<br>Met (1)	Cd (1)<br>Mor (1)<br>Met (1)
T8	Cd (1)	Cd (1)	Cd (1)	Cd (1)	Cd (1)	Cd (1)	Cd (1)

Time	0 µg/L	0.05 µg/L	0.2 µg/L	1 µg/L	3.5 µg/L	7 µg/L	70 µg/L
	Mor (1)<br>Met (1)<br>Ele (7)	Mor (1)<br>Met (1)<br>Ele (7)	Mor (1)<br>Met (1)<br>Ele (7)	Mor (1)<br>Met (1)<br>Ele (7)	Mor (1)<br>Met (1)<br>Ele (7)	Mor (1)<br>Met (1)<br>Ele (7)	Mor (1)<br>Met (1)<br>Ele (7)

Notes: Cd: Cd concentration in sediment, Mor: Morphological analysis, Met: Metabarcoding, Ele: Elemental composition of foraminiferal tests. The numbers in parentheses indicate the number of culture chambers.

Table S2. Relative abundance (%) of benthic foraminiferal orders over time and under different Cd concentrations, based on eDNA metabarcoding.

Cd concentration	Time	Allogromida	Astrorhizida	Lagenina	Lituolida	Miliolida	Rotaliida	Textulariida	unclassified
0 µg/L	T0	1.87	10.77	0.74	0.54	10.75	59.15	2.84	13.34
	T1	0.61	2.55	0.14	0.18	8.48	66.35	1.77	19.91
	T2	0.99	1.53	1.26	4.30	14.71	62.10	1.70	13.41
	T3	0.40	3.46	0.48	0.84	8.42	80.47	0.37	5.56
	T4	0.00	1.15	0.02	0.19	5.42	92.36	0.03	0.84
	T6	0.01	1.50	0.49	3.07	53.74	30.07	3.74	7.39
	T8	0.12	0.54	0.02	0.32	86.55	11.95	0.06	0.44
0.05 µg/L	T0	1.87	10.77	0.74	0.54	10.75	59.15	2.84	13.34
	T1	0.08	4.14	1.55	0.75	10.99	65.05	1.46	15.98
	T2	0.00	4.24	2.38	0.19	17.77	64.18	0.52	10.72
	T3	0.20	3.77	0.39	2.15	19.13	62.40	0.47	11.49
	T4	0.05	0.60	0.01	5.88	3.10	21.42	0.01	68.93
	T6	0.00	0.45	0.19	2.64	59.43	27.55	0.08	9.65
	T8	0.01	0.06	0.02	0.11	13.92	85.26	0.02	0.60
0.2 µg/L	T0	1.87	10.77	0.74	0.54	10.75	59.15	2.84	13.34
	T1	0.16	5.09	0.01	0.03	15.69	64.24	1.57	13.21
	T2	0.00	3.69	0.06	4.08	8.48	72.84	0.29	10.56
	T3	0.73	7.84	0.00	2.73	6.23	73.24	1.77	7.46

	T4	0.17	3.33	0.32	1.35	32.89	39.96	8.52	13.45
	T6	0.02	1.25	0.03	1.82	4.98	90.74	0.01	1.16
	T8	0.05	0.38	0.13	1.74	81.59	14.87	0.21	1.03
1 μg/L	T0	1.87	10.77	0.74	0.54	10.75	59.15	2.84	13.34
	T1	0.81	2.57	0.04	1.18	10.46	64.46	2.12	18.37
	T2	0.00	7.72	0.00	3.68	13.28	70.38	0.58	4.37
	T3	0.16	2.49	0.06	4.46	10.22	55.66	3.88	23.06
	T4	0.02	0.32	0.60	1.98	21.78	67.16	0.46	7.68
	T6	0.00	0.25	0.03	0.82	6.65	90.80	0.03	1.42
	T8	0.19	2.98	0.42	6.94	14.03	61.77	0.52	13.16
5 μg/L	T0	1.87	10.77	0.74	0.54	10.75	59.15	2.84	13.34
	T1	0.59	3.85	0.37	0.45	13.66	65.31	1.25	14.51
	T2	0.00	2.28	0.65	0.56	18.31	71.04	0.65	6.51
	T3	0.03	1.49	0.16	2.51	19.76	62.10	0.65	13.31
	T4	0.03	7.83	0.01	0.25	88.17	3.40	0.00	0.31
	T6	0.06	0.21	0.08	5.35	65.76	27.65	0.01	0.87
	T8	0.00	0.21	0.03	0.64	23.80	60.06	11.21	4.04
10 μg/L	T0	1.87	10.77	0.74	0.54	10.75	59.15	2.84	13.34
	T1	0.42	3.67	0.11	0.37	11.23	70.28	1.69	12.24
	T2	0.00	3.07	1.69	0.16	9.44	73.89	1.93	9.81
	T3	0.05	0.20	0.05	0.23	1.81	6.98	0.23	90.45
	T4	0.05	8.76	0.22	0.98	54.88	29.62	0.12	5.37
	T6	0.08	1.54	0.20	4.20	37.24	53.01	0.31	3.41
	T8	0.01	0.38	0.09	2.02	72.73	20.86	0.22	3.69
100 μg/L	T0	1.87	10.77	0.74	0.54	10.75	59.15	2.84	13.34
	T1	0.07	3.83	0.24	4.63	12.11	56.13	2.15	20.84
	T2	0.26	14.37	0.49	10.59	9.04	56.51	1.01	7.73
	T3	0.01	0.26	0.00	0.94	93.94	4.35	0.04	0.46
	T4	0.01	2.46	0.00	12.39	11.49	67.42	0.41	5.84
	T6	0.42	4.22	0.01	22.65	17.49	50.48	0.77	3.96

	T8	0.23	2.15	0.00	19.42	7.54	54.64	0.28	15.75
Average	T0	1.87	10.77	0.74	0.54	10.75	59.15	2.84	13.34
	T1	0.39	3.67	0.35	1.09	11.80	64.54	1.72	16.44
	T2	0.18	5.27	0.93	3.37	13.00	67.28	0.96	9.01
	T3	0.23	2.79	0.16	1.98	22.79	49.31	1.06	21.68
	T4	0.05	3.49	0.17	3.29	31.10	45.91	1.37	14.63
	T6	0.09	1.35	0.15	5.79	35.04	52.90	0.71	3.98
	T8	0.09	0.96	0.10	4.46	42.88	44.20	1.79	5.53

Table S3. Statistical results (eta-squared and p-values) for foraminiferal orders and genera based on DNA and RNA datasets. Significant p-values (<0.05) are shown in bold.

Level	Taxon	eDNA		eRNA	
		η^2	*p* value	η^2	*p* value
Order	Allogromida	0.11	0.637	0.129	0.531
	Astrorhizida	0.09	0.745	**0.37**	**0.009**
	Lagenina	0.192	0.249	0.117	0.597
	Lituolida	**0.436**	**0.002**	**0.304**	**0.038**
	Miliolida	0.086	0.768	0.059	0.895
	Rotaliida	0.1	0.693	0.100	0.689
	Textulariida	0.077	0.812	0.152	0.415
Genus	*Ammobaculites*	0.103	0.673	0.173	0.318
	Ammonia	0.158	0.385	0.097	0.706
	Crithionina	0.014	0.998	0.146	0.440
	Elphidium	0.123	0.564	0.127	0.542
	Epistominella	0.144	0.454	0.123	0.566
	Flexammina	0.287	0.052	0.146	0.441
	Glabratella	0.054	0.913	0.096	0.716
	Miliammina	0.119	0.583	**0.328**	**0.023**
	Neoassilina	0.06	0.893	0.076	0.821
	Notodendrodes	0.132	0.515	0.174	0.314

Nummulites	0.235	0.130	0.132	0.516
Operculina	0.128	0.538	0.123	0.564
Ovammina	0.058	0.902	0.093	0.730
Parasorites	0.015	0.997	0.136	0.492
Pulleniatina	0.11	0.637	0.143	0.456
Quinqueloculina	0.087	0.763	0.054	0.915
Reophax	**0.54**	**0.000**	**0.315**	**0.030**
Uvigerina	0.155	0.400	0.155	0.397

Table S4. Relative abundance (%) of benthic foraminiferal genera over time and under different Cd concentrations, based on eDNA metabarcoding.

Cd concentration	Time	*Ammobaculites*	*Ammonia*	*Crithionina*	*Elphidium*	*Epistominella*	*Flexammina*	*Glabratella*	*Miliammina*	*Neoassilina*	*Notodendrodes*	*Nummulites*	*Operculina*	*Ovammina*	*Parasorites*	*Pullenia*	*Pulleniatina*	*Quinqueloculina*	*Reophax*	*Uvigerina*	Others
	T0	0.00	0.00	13.21	0.00	0.00	0.00	3.94	0.00	18.11	0.00	12.17	19.78	10.61	8.88	0.00	0.53	0.00	0.54	2.15	10.07
	T1	0.00	1.21	18.09	0.05	0.00	0.00	7.24	0.13	19.28	0.00	13.32	22.57	2.26	7.77	0.00	0.17	0.00	0.18	0.60	7.13
	T2	0.00	9.59	11.93	0.04	0.00	0.00	9.25	0.66	15.36	0.00	8.32	15.38	1.39	11.52	0.32	0.27	0.00	4.30	1.68	9.99
0 µg/L	T3	0.00	2.05	3.94	0.05	0.00	0.00	8.01	0.31	7.64	0.00	45.88	13.83	3.04	5.90	0.00	0.19	0.96	0.84	0.78	6.58
	T4	0.00	0.10	0.34	0.00	0.00	0.00	0.71	0.50	0.83	0.00	88.99	1.29	1.08	0.36	0.00	0.00	4.95	0.19	0.01	0.65
	T6	2.72	0.11	3.09	0.00	0.61	0.00	3.60	2.93	5.39	0.98	6.83	13.08	1.05	2.20	0.00	0.11	51.46	3.07	0.15	2.60
	T8	0.00	2.77	0.41	0.27	0.04	0.00	0.40	0.03	2.06	0.00	1.02	4.88	0.37	1.77	0.00	0.04	84.62	0.32	0.33	0.68
0.05 µg/L	T0	0.00	0.00	13.21	0.00	0.00	0.00	3.94	0.00	18.11	0.00	12.17	19.78	10.61	8.88	0.00	0.53	0.00	0.54	2.15	10.07
	T1	0.00	1.66	15.60	0.02	0.00	0.00	8.07	0.00	21.88	0.00	6.71	17.97	3.41	9.33	0.00	0.14	0.00	0.75	3.43	11.02

	T2	0.00	0.00	6.95	0.00	0.00	0.00	10.12	2.92	13.68	0.00	9.25	29.06	3.90	17.53	0.00	0.16	0.11	0.19	1.22	4.90
	T3	0.00	1.89	8.85	0.00	0.05	0.00	6.14	0.45	25.87	0.00	5.97	14.39	1.72	7.00	0.00	0.36	11.74	2.15	1.32	12.10
	T4	0.00	1.74	1.20	0.00	0.00	0.00	2.87	67.27	4.16	0.00	6.74	5.66	0.39	2.52	0.00	0.03	0.09	5.88	0.10	1.38
	T6	0.00	5.15	1.23	10.97	0.00	0.00	1.92	8.36	1.79	0.00	3.53	2.47	0.25	1.36	0.00	0.07	57.55	2.64	0.33	2.36
	T8	0.00	83.59	0.09	0.00	0.00	0.00	0.18	0.39	0.29	0.03	0.20	0.45	0.06	0.33	0.00	0.00	13.53	0.11	0.01	0.73
	T0	0.00	0.00	13.21	0.00	0.00	0.00	3.94	0.00	18.11	0.00	12.17	19.78	10.61	8.88	0.00	0.53	0.00	0.54	2.15	10.07
	T1	0.00	1.58	11.69	0.05	0.00	0.00	4.74	0.58	27.96	0.00	7.03	18.34	3.74	15.21	0.00	1.36	0.01	0.03	1.10	6.59
	T2	0.00	0.00	9.90	0.00	0.00	0.00	17.70	0.00	25.55	0.00	8.31	19.77	3.60	7.72	0.00	0.22	0.00	4.08	0.47	2.69
0.2 µg/L	T3	0.00	0.00	6.91	0.00	0.00	0.00	8.47	0.00	17.73	0.00	29.19	14.83	2.19	4.75	0.00	0.40	0.74	2.73	0.34	11.71
	T4	5.55	3.51	5.24	0.04	0.00	0.00	3.31	7.41	14.24	0.00	7.16	10.72	3.33	2.45	0.00	0.18	30.24	1.35	0.05	5.22
	T6	0.00	0.26	0.19	2.54	0.00	0.00	0.40	0.06	0.29	0.00	1.32	85.76	0.14	2.60	0.00	0.00	2.31	1.82	0.01	2.30
	T8	0.00	5.12	0.67	0.84	0.00	0.00	0.40	0.32	1.91	0.02	0.98	4.71	0.19	1.26	0.00	0.00	80.21	1.74	0.06	1.57
	T0	0.00	0.00	13.21	0.00	0.00	0.00	3.94	0.00	18.11	0.00	12.17	19.78	10.61	8.88	0.00	0.53	0.00	0.54	2.15	10.07
	T1	0.00	0.01	17.57	0.00	0.00	0.00	6.75	0.12	19.70	0.00	9.32	24.05	2.38	9.05	0.00	0.29	0.40	1.18	1.06	8.12
	T2	0.00	7.07	2.71	0.00	0.00	0.00	7.94	1.02	17.73	0.00	6.97	9.89	6.96	6.74	14.84	0.15	0.22	3.68	0.00	14.08
1 µg/L	T3	2.75	0.00	5.90	0.00	0.00	0.00	5.19	16.26	24.99	0.00	7.25	14.95	2.46	8.26	0.00	0.16	0.90	4.46	0.96	5.52
	T4	0.00	1.39	3.94	0.43	1.44	0.00	12.34	2.17	15.65	0.00	12.34	20.58	0.32	10.04	0.00	0.07	11.50	1.98	0.26	5.54
	T6	0.00	87.86	0.12	0.00	0.09	0.00	0.24	1.26	0.52	0.00	0.63	1.30	0.05	0.13	0.00	0.01	0.93	0.82	0.02	6.03

	T8	0.00	17.45	4.81	1.34	0.00	0.00	4.02	1.14	14.57	0.19	9.37	13.17	1.01	2.33	0.00	0.06	7.63	6.94	0.09	15.88
5 µg/L	T0	0.00	0.00	13.21	0.00	0.00	0.00	3.94	0.00	18.11	0.00	12.17	19.78	10.61	8.88	0.00	0.53	0.00	0.54	2.15	10.07
	T1	0.00	1.04	13.71	0.10	0.09	0.00	5.18	0.40	19.97	0.00	11.60	22.27	3.82	9.30	0.00	0.16	0.80	0.45	1.22	9.89
	T2	0.00	0.01	5.82	0.00	0.00	0.00	8.55	0.19	25.20	0.00	18.31	14.07	2.05	11.63	0.00	0.67	5.37	0.56	0.07	7.50
	T3	0.00	0.69	11.86	0.03	0.08	0.00	6.52	1.00	24.12	0.00	14.78	13.88	0.85	8.93	0.00	0.29	10.66	2.51	0.09	3.72
	T4	0.00	0.02	0.23	0.00	0.00	0.00	0.19	0.07	0.45	0.00	1.69	0.93	0.09	0.20	0.00	0.00	87.87	0.25	0.05	7.96
	T6	0.00	0.91	0.74	0.06	0.00	0.00	1.09	0.10	3.64	0.00	15.15	6.29	0.20	1.25	0.00	0.06	64.18	5.35	0.02	0.96
	T8	11.03	5.07	0.28	0.36	47.43	0.00	0.68	3.65	2.42	0.00	0.98	2.31	0.11	0.93	0.00	0.00	22.86	0.64	0.00	1.25
10 µg/L	T0	0.00	0.00	13.21	0.00	0.00	0.00	3.94	0.00	18.11	0.00	12.17	19.78	10.61	8.88	0.00	0.53	0.00	0.54	2.15	10.07
	T1	0.00	0.02	11.87	0.15	0.00	0.00	7.38	0.16	21.18	0.00	13.66	23.22	3.05	9.61	0.00	1.10	0.04	0.37	1.38	6.82
	T2	0.00	0.37	8.38	0.26	0.00	0.00	8.75	1.00	22.87	0.00	11.22	24.30	3.07	7.49	0.63	0.18	1.39	0.16	0.00	9.94
	T3	0.10	0.06	0.74	0.00	0.07	0.00	0.79	89.70	1.45	0.00	1.36	2.94	0.18	1.13	0.00	0.02	0.57	0.23	0.02	0.63
	T4	0.00	1.01	4.66	0.07	0.00	0.00	1.57	0.63	8.15	0.00	4.82	11.55	1.33	5.06	0.00	0.20	42.35	0.98	0.29	17.33
	T6	0.00	0.47	2.86	0.00	0.00	0.03	2.56	0.38	8.59	0.00	4.38	35.44	1.05	3.02	0.00	0.06	31.52	4.20	0.04	5.42
	T8	0.00	4.50	1.98	0.02	0.00	0.02	0.79	0.73	6.13	0.00	2.05	5.37	0.37	2.49	0.00	0.03	70.03	2.02	0.05	3.42
100 µg/L	T0	0.00	0.00	13.21	0.00	0.00	0.00	3.94	0.00	18.11	0.00	12.17	19.78	10.61	8.88	0.00	0.53	0.00	0.54	2.15	10.07
	T1	0.00	0.00	19.75	0.21	0.01	0.00	6.05	0.76	16.67	0.00	8.19	22.44	2.96	10.74	0.00	0.18	0.00	4.63	1.29	6.11
	T2	0.00	0.54	6.31	0.00	0.00	0.00	5.51	0.47	20.07	0.00	6.07	21.90	2.08	8.04	0.00	0.12	0.26	10.59	0.39	17.66

	T3	0.00	0.08	0.43	0.00	0.21	0.00	0.37	0.02	1.85	0.00	0.70	0.88	0.21	0.88	0.00	0.01	92.51	0.94	0.06	0.83
	T4	0.00	6.88	1.26	0.00	0.00	0.00	2.69	4.42	9.99	0.05	6.92	40.08	0.52	2.16	0.00	0.08	3.05	12.39	0.45	9.07
	T6	0.00	3.76	3.68	0.00	1.43	0.00	3.92	0.17	13.57	0.00	4.53	18.45	0.82	3.49	0.00	0.11	12.27	22.65	0.66	10.49
	T8	0.00	5.49	11.74	0.00	0.00	0.00	6.41	3.81	12.83	0.00	9.34	14.64	1.63	5.34	0.00	0.04	0.37	19.42	0.24	8.72
Average	T0	0.00	0.00	13.21	0.00	0.00	0.00	3.94	0.00	18.11	0.00	12.17	19.78	10.61	8.88	0.00	0.53	0.00	0.54	2.15	10.07
	T1	0.00	0.79	15.47	0.08	0.01	0.00	6.49	0.31	20.95	0.00	9.98	21.55	3.09	10.14	0.00	0.48	0.18	1.09	1.44	7.95
	T2	0.00	2.51	7.43	0.04	0.00	0.00	9.69	0.89	20.07	0.00	9.78	19.19	3.29	10.10	2.26	0.25	1.05	3.37	0.55	9.54
	T3	0.41	0.68	5.52	0.01	0.06	0.00	5.07	15.39	14.81	0.00	15.02	10.81	1.52	5.27	0.00	0.21	16.87	1.98	0.51	5.87
	T4	0.79	2.09	2.41	0.08	0.21	0.00	3.38	11.78	7.64	0.01	18.38	12.97	1.01	3.25	0.00	0.08	25.72	3.29	0.17	6.73
	T6	0.39	14.08	1.70	1.94	0.30	0.00	1.96	1.89	4.83	0.14	5.20	23.26	0.51	2.01	0.00	0.06	31.46	5.79	0.18	4.31
	T8	1.58	17.71	2.85	0.40	6.78	0.00	1.84	1.44	5.75	0.03	3.42	6.50	0.54	2.06	0.00	0.03	39.89	4.46	0.11	4.60

Table S5. Relative abundance (%) of benthic foraminiferal orders over time and under different Cd concentrations, based on eRNA metabarcoding.

Cd concentration	Time	Allogromida	Astrorhizida	Lagenina	Lituolida	Miliolida	Rotaliida	Textulariida	unclassified
0 μg/L	T0	0.00	43.49	0.00	33.41	23.09	0.00	0.00	0.00
	T1	4.44	0.66	0.00	0.35	1.85	89.92	1.07	1.71
	T2	0.00	0.00	0.00	0.00	99.07	0.93	0.00	0.00
	T3	1.84	1.13	0.22	8.15	67.19	11.77	1.95	7.75
	T4	0.00	4.66	0.00	0.15	4.55	89.34	0.00	1.30
	T6	0.00	0.02	0.00	2.98	22.70	7.31	0.11	66.89
	T8	0.15	0.00	0.00	4.80	18.14	67.46	0.89	8.55
0.05 μg/L	T0	0.00	43.49	0.00	33.41	23.09	0.00	0.00	0.00

	T1	0.04	2.35	0.16	9.32	10.80	75.88	0.88	0.57
	T2	0.00	0.21	0.00	8.99	60.41	26.86	0.01	3.53
	T3	0.00	0.00	0.00	0.00	83.01	8.94	0.00	8.05
	T4	0.00	15.60	0.00	0.00	20.47	34.54	0.00	29.38
	T6	7.10	27.30	0.00	9.38	30.62	15.71	0.00	9.88
	T8	0.27	5.53	0.00	3.62	4.38	53.60	0.01	32.58
0.2 μg/L	T0	0.00	43.49	0.00	33.41	23.09	0.00	0.00	0.00
	T1	0.00	1.64	1.77	4.01	47.90	35.99	6.63	2.05
	T2	0.00	0.00	0.00	38.38	1.74	47.59	1.33	10.96
	T3	0.00	4.09	0.00	0.00	89.59	3.55	0.66	2.11
	T4	0.00	0.00	0.00	4.62	18.10	53.71	0.35	23.21
	T6	0.00	0.49	0.00	0.00	39.01	57.69	0.00	2.81
	T8	0.31	0.00	0.00	2.79	82.14	13.91	0.08	0.77
1 μg/L	T0	0.00	43.49	0.00	33.41	23.09	0.00	0.00	0.00
	T1	0.00	0.00	0.00	0.02	0.01	27.66	0.00	72.32
	T2	0.05	0.00	0.00	56.65	12.82	22.52	0.00	7.97
	T3	0.00	0.00	0.00	1.65	83.94	7.66	3.59	3.16
	T4	0.00	0.00	0.00	0.94	90.83	2.80	0.00	5.42
	T6	0.00	0.44	1.00	0.00	38.63	58.33	0.00	1.59
	T8	0.00	0.00	0.00	15.05	31.38	52.52	0.43	0.62
5 μg/L	T0	0.00	43.49	0.00	33.41	23.09	0.00	0.00	0.00
	T1	0.00	0.00	0.00	10.58	0.37	64.78	0.00	24.27
	T2	0.00	0.00	0.00	24.32	56.84	12.76	4.12	1.97
	T3	0.00	0.00	0.00	0.49	89.80	6.64	2.26	0.81
	T4	0.00	0.00	0.00	2.88	80.11	8.34	0.00	8.67
	T6	0.18	0.00	0.00	4.62	82.21	12.57	0.00	0.43
	T8	0.00	2.22	0.00	5.60	13.84	44.42	32.92	1.00
10 μg/L	T0	0.00	43.49	0.00	33.41	23.09	0.00	0.00	0.00
	T1	0.80	4.63	0.00	28.04	6.98	50.94	7.49	1.12
	T2	0.00	0.00	0.00	41.49	20.39	22.67	0.00	15.44

	T3	8.60	0.00	0.00	6.46	74.97	2.59	7.23	0.15
	T4	0.00	0.00	0.00	1.14	83.28	14.46	0.01	1.11
	T6	0.00	0.00	0.00	9.41	67.41	7.13	0.00	16.05
	T8	0.00	0.00	0.00	0.00	89.28	10.72	0.00	0.00
100 μg/L	T0	0.00	43.49	0.00	33.41	23.09	0.00	0.00	0.00
	T1	0.00	0.00	0.00	0.00	99.93	0.07	0.00	0.00
	T2	0.02	0.26	0.00	28.94	23.47	36.71	3.89	6.72
	T3	0.00	0.00	0.00	45.39	53.23	0.22	0.00	1.16
	T4	0.00	0.15	0.00	59.98	12.89	26.51	0.35	0.12
	T6	0.00	1.42	0.00	28.71	5.57	64.20	0.00	0.11
	T8	0.00	0.10	0.00	41.04	3.59	51.56	0.06	3.65
Average	T0	0.00	43.49	0.00	33.41	23.09	0.00	0.00	0.00
	T1	0.75	1.33	0.28	7.47	23.98	49.32	2.30	14.58
	T2	0.01	0.07	0.00	28.39	39.25	24.29	1.33	6.66
	T3	1.49	0.75	0.03	8.88	77.39	5.91	2.24	3.31
	T4	0.00	2.92	0.00	9.96	44.32	32.82	0.10	9.89
	T6	1.04	4.24	0.14	7.87	40.88	31.85	0.02	13.96
	T8	0.10	1.12	0.00	10.42	34.68	42.03	4.91	6.74

Table S6. Relative abundance (%) of benthic foraminiferal genera over time and under different Cd concentrations, based on eRNA metabarcoding.

Cd concentration	Time	*Ammobaculites*	*Ammonia*	*Crithionina*	*Elphidium*	*Epistominella*	*Flexammina*	*Glabratella*	*Miliammina*	*Neoassilina*	*Notodendrodes*	*Nummulites*	*Operculina*	*Ovammina*	*Parasorites*	*Pullenia*	*Pulleniatina*	*Quinqueloculina*	*Reophax*	*Uvigerina*	Others
	T0	0.00	0.00	0.00	0.00	0.00	0.00	0.00	0.00	0.00	0.00	0.00	0.00	43.49	23.09	0.00	0.00	0.00	33.41	0.00	0.00
0 μg/L	T1	0.00	0.00	0.29	0.00	0.00	0.00	25.10	0.99	27.99	0.00	6.78	26.33	0.66	1.32	0.00	0.00	0.00	0.35	0.00	10.19
	T2	0.00	0.00	0.00	0.00	0.00	0.00	0.00	0.00	0.00	0.00	0.86	0.06	0.00	0.00	0.00	0.00	99.07	0.00	0.00	0.00

	T3	0.00	1.52	0.39	0.00	0.00	0.00	0.00	7.36	1.42	0.00	1.12	2.41	1.13	0.01	0.00	0.00	66.95	8.15	0.00	9.55
	T4	0.00	0.05	0.00	0.00	0.00	0.00	0.00	1.03	0.00	0.00	89.17	0.00	4.66	0.00	0.00	0.00	4.55	0.15	0.00	0.39
	T6	0.11	2.44	0.00	0.00	2.60	0.00	0.00	3.02	0.90	63.87	0.39	0.05	0.02	0.00	0.00	0.00	22.70	2.98	0.00	0.93
	T8	0.00	59.44	0.00	1.38	5.11	0.00	0.00	0.78	0.00	7.76	0.26	0.61	0.00	0.00	0.00	0.00	18.13	4.80	0.00	1.71
0.05 µg/L	T0	0.00	0.00	0.00	0.00	0.00	0.00	0.00	0.00	0.00	0.00	0.00	0.00	43.49	23.09	0.00	0.00	0.00	33.41	0.00	0.00
	T1	0.00	0.07	0.18	0.02	0.29	0.00	15.46	0.39	27.17	0.00	6.67	19.12	2.23	9.99	0.00	0.02	0.00	9.32	0.43	8.65
	T2	0.00	2.57	0.00	0.00	0.01	0.00	2.82	3.53	0.00	0.00	1.93	0.84	0.21	0.00	0.00	0.00	60.32	8.99	0.00	18.78
	T3	0.00	0.00	0.00	0.00	0.00	0.00	0.00	8.05	0.00	0.00	0.00	0.00	0.00	0.00	0.00	0.00	83.01	0.00	0.00	8.93
	T4	0.00	0.00	0.00	0.00	0.00	0.00	0.00	29.38	0.00	0.00	21.57	0.00	0.00	0.00	0.00	0.00	20.47	0.00	12.97	15.60
	T6	0.00	6.94	0.00	0.00	0.00	0.00	0.00	9.88	0.00	0.00	3.56	0.00	0.00	0.00	0.00	0.00	30.59	9.38	0.00	39.65
	T8	0.00	27.97	0.00	0.00	8.89	0.00	0.00	25.92	0.39	6.66	0.04	0.00	0.00	0.00	0.00	0.00	4.38	3.62	0.00	22.12
0.2 µg/L	T0	0.00	0.00	0.00	0.00	0.00	0.00	0.00	0.00	0.00	0.00	0.00	0.00	43.49	23.09	0.00	0.00	0.00	33.41	0.00	0.00
	T1	0.00	8.26	1.51	0.39	0.00	0.00	2.43	0.00	3.12	0.00	11.60	7.57	1.16	1.60	0.00	0.16	46.02	4.01	0.00	12.17
	T2	0.00	17.39	0.23	0.50	0.00	0.00	0.15	10.54	9.18	0.00	2.92	1.50	0.00	0.17	0.00	0.08	1.36	38.38	0.00	17.59
	T3	0.00	0.00	2.11	0.00	0.00	0.00	0.00	0.00	0.00	0.00	3.55	0.00	4.09	0.00	0.00	0.00	83.81	0.00	0.00	6.44
	T4	0.00	0.13	0.00	0.00	0.00	0.00	0.00	23.21	0.00	0.00	0.01	53.57	0.00	3.08	0.00	0.00	15.02	4.62	0.00	0.35
	T6	0.00	1.17	1.44	32.12	8.02	0.00	0.00	1.37	0.00	0.00	1.00	14.33	0.00	0.00	0.00	0.00	39.01	0.00	0.00	1.55
	T8	0.00	9.19	0.00	0.08	4.09	0.00	0.00	0.68	0.00	0.09	0.03	0.22	0.00	0.00	0.00	0.00	82.14	2.79	0.00	0.70

1 µg/L	T0	0.00	0.00	0.00	0.00	0.00	0.00	0.00	0.00	0.00	0.00	0.00	0.00	43.49	23.09	0.00	0.00	0.00	33.41	0.00	0.00
	T1	0.00	0.01	72.32	0.00	0.00	0.00	0.00	0.00	0.00	0.00	0.00	0.01	0.00	0.00	0.00	27.64	0.01	0.02	0.00	0.00
	T2	0.00	7.09	0.00	0.00	0.02	0.00	0.00	7.97	0.00	0.00	3.83	11.55	0.00	0.00	0.00	0.00	11.32	56.65	0.00	1.57
	T3	3.59	7.16	2.31	0.00	0.00	0.00	0.00	0.85	0.00	0.00	0.00	0.00	0.00	0.00	0.00	0.00	83.94	1.65	0.00	0.49
	T4	0.00	2.65	0.00	0.00	0.00	0.00	0.00	5.42	0.00	0.00	0.00	0.00	0.00	0.00	0.00	0.00	90.83	0.94	0.00	0.15
	T6	0.00	3.74	0.97	1.97	16.12	0.00	0.00	0.40	0.00	0.00	0.86	35.61	0.44	0.00	0.00	0.03	37.77	0.00	0.00	2.08
	T8	0.43	28.62	0.00	22.12	0.00	0.00	0.00	0.62	1.04	0.00	0.00	0.00	0.00	0.00	0.00	0.00	29.30	15.05	0.00	2.81
5 µg/L	T0	0.00	0.00	0.00	0.00	0.00	0.00	0.00	0.00	0.00	0.00	0.00	0.00	43.49	23.09	0.00	0.00	0.00	33.41	0.00	0.00
	T1	0.00	0.00	7.71	0.00	0.00	0.00	16.67	7.09	8.94	0.00	26.69	12.45	0.00	0.37	0.00	0.00	0.00	10.58	0.00	9.50
	T2	4.12	4.40	0.08	0.00	0.01	0.00	0.56	1.11	0.00	0.00	2.51	0.37	0.00	0.00	0.00	0.02	56.84	24.32	0.00	5.68
	T3	0.00	0.00	0.56	0.00	0.00	0.00	0.00	0.00	0.00	0.00	3.82	0.00	0.00	0.00	0.00	0.00	89.80	0.49	0.00	5.34
	T4	0.00	0.00	0.00	0.00	0.00	0.00	0.00	6.71	0.00	0.00	6.49	0.00	0.00	0.00	0.00	1.86	80.11	2.88	0.00	1.96
	T6	0.00	0.00	0.00	2.68	5.76	0.00	0.00	0.43	0.00	0.00	0.00	3.19	0.00	0.00	0.00	0.00	82.21	4.62	0.00	1.12
	T8	32.92	6.53	0.00	5.34	30.28	0.00	0.00	1.00	1.02	0.00	0.00	0.91	2.22	0.00	0.00	0.00	13.84	5.60	0.00	0.33
10 µg/L	T0	0.00	0.00	0.00	0.00	0.00	0.00	0.00	0.00	0.00	0.00	0.00	0.00	43.49	23.09	0.00	0.00	0.00	33.41	0.00	0.00
	T1	0.00	22.96	1.11	0.00	1.42	0.00	2.42	0.01	9.15	0.00	6.21	5.71	4.63	1.46	0.00	0.00	0.02	28.04	0.00	16.87
	T2	0.00	0.03	0.00	0.00	0.01	0.00	3.33	15.44	0.00	0.00	0.00	8.26	0.00	0.00	0.00	0.00	16.75	41.49	0.00	14.67
	T3	0.00	0.00	0.00	0.00	0.00	0.00	0.00	0.15	0.00	0.00	2.59	0.00	0.00	24.46	0.00	0.00	50.51	6.46	0.00	15.83

	T4	0.00	0.83	0.47	0.00	12.91	0.00	0.00	0.65	0.00	0.00	0.01	0.00	0.00	0.00	0.00	0.00	83.28	1.14	0.00	0.72
	T6	0.00	4.02	0.00	0.00	0.00	10.88	0.00	2.87	0.56	2.31	0.00	0.83	0.00	0.00	0.00	0.00	67.29	9.41	0.00	1.84
	T8	0.00	10.18	0.00	0.00	0.00	0.00	0.00	0.00	0.00	0.00	0.55	0.00	0.00	0.00	0.00	0.00	89.28	0.00	0.00	0.00
100 µg/L	T0	0.00	0.00	0.00	0.00	0.00	0.00	0.00	0.00	0.00	0.00	0.00	0.00	43.49	23.09	0.00	0.00	0.00	33.41	0.00	0.00
	T1	0.00	0.00	0.00	0.02	0.01	0.00	0.00	0.00	0.00	0.00	0.00	0.03	0.00	0.00	0.00	0.02	99.41	0.00	0.00	0.52
	T2	3.63	28.82	0.07	0.00	0.00	0.00	0.64	6.65	3.41	0.00	0.24	0.91	0.00	0.00	0.00	0.00	15.89	28.94	0.00	10.82
	T3	0.00	0.00	1.16	0.00	0.00	0.00	0.00	0.00	0.00	0.00	0.00	0.00	0.00	0.00	0.00	0.00	52.84	45.39	0.00	0.61
	T4	0.00	21.63	0.00	0.25	0.00	0.00	0.00	0.12	0.28	0.00	1.05	1.81	0.15	0.03	0.00	0.02	8.70	59.98	0.00	5.98
	T6	0.00	9.52	0.00	0.00	51.10	0.00	0.00	0.00	1.31	0.11	0.00	0.00	0.00	0.02	0.00	0.00	1.31	28.71	0.00	7.93
	T8	0.06	33.90	3.64	0.00	16.59	0.00	0.00	0.02	0.18	0.00	0.32	0.44	0.10	0.00	0.00	0.00	2.70	41.04	0.00	1.01
Average	T0	0.00	0.00	0.00	0.00	0.00	0.00	0.00	0.00	0.00	0.00	0.00	0.00	43.49	23.09	0.00	0.00	0.00	33.41	0.00	0.00
	T1	0.00	4.47	11.87	0.06	0.25	0.00	8.87	1.21	10.91	0.00	8.28	10.17	1.24	2.11	0.00	3.98	20.78	7.47	0.06	8.27
	T2	1.11	8.61	0.05	0.07	0.01	0.00	1.07	6.46	1.80	0.00	1.76	3.36	0.03	0.02	0.00	0.01	37.37	28.39	0.00	9.87
	T3	0.51	1.24	0.93	0.00	0.00	0.00	0.00	2.34	0.20	0.00	1.58	0.35	0.75	3.50	0.00	0.00	72.98	8.88	0.00	6.74
	T4	0.00	3.61	0.07	0.04	1.84	0.00	0.00	9.50	0.04	0.00	16.90	7.91	0.69	0.44	0.00	0.27	43.28	9.96	1.85	3.59
	T6	0.02	3.98	0.34	5.25	11.94	1.55	0.00	2.57	0.40	9.47	0.83	7.72	0.07	0.00	0.00	0.00	40.12	7.87	0.00	7.87
	T8	4.77	25.12	0.52	4.13	9.28	0.00	0.00	4.15	0.38	2.07	0.17	0.31	0.33	0.00	0.00	0.00	34.25	10.42	0.00	4.10
